# Preliminary study on miRNA in prostate cancer

**DOI:** 10.1186/s12957-023-03151-1

**Published:** 2023-08-29

**Authors:** Saimaitikari Abudoubari, Ke Bu, Yujie Mei, Abudukeyoumu Maimaitiyiming, Hengqing An, Ning Tao

**Affiliations:** 1 College of Public Health, Xinjiang Medical University, Urumqi, 830011 Xinjiang China; 2 Department of Radiology, The First People’s Hospital of Kashi Prefecture, Kashi, 844700 Xinjiang China; 3 The First Affiliated Hospital, Xinjiang Medical University, No. 393, Xinyi Road, Xinshi District, Urumqi, 830011 Xinjiang China; 4 Xinjiang Clinical Research Center for Genitourinary System, No. 393, Xinyi Road, Xinshi District, Urumqi, 830011 Xinjiang China

**Keywords:** Prostate cancer, miRNA, Chip technology

## Abstract

**Objective:**

To screen for miRNAs differentially expressed in prostate cancer and prostate hyperplasia tissues and to validate their association with prostate cancer.

**Methods:**

Patients diagnosed by pathology in the Department of Urology of the First Affiliated Hospital of Xinjiang Medical University from October 2021 to June 2022 were selected, and their general clinical information, blood samples, and prostate tissue samples were collected. miRNA microarray technology was performed to obtain differentially expressed miRNAs in prostate cancer and hyperplasia tissues, and miRNAs to be studied were screened by microarray results and review of relevant literature. The detection of miRNA expression in the patients’ blood and prostate tissue samples was measured. The miRNA-222-mimics were transfected into PC3 cells, and cell biology experiments such as CCK8, scratch, Transwell, and flow cytometry were performed to detect the effects of overexpressed miRNA-222 on the growth and proliferation, invasive ability, apoptotic ability, and metastatic ability of prostate cancer cells.

**Results:**

The results of the miRNA microarray showed that there were many differentially expressed miRNAs in prostate cancer and hyperplasia tissues, and four miRNAs, miRNA-144, miRNA-222, miRNA-1248, and miRNA-3651 were finally selected as the subjects by reviewing relevant literature. The results showed that the expression of miRNA-222 in prostate cancer tissues was lower than that in prostate hyperplasia tissues (*P* < 0.05). The expression of miRNA-222, miRNA-1248, and miRNA-3651 in blood samples of prostate cancer patients was lower than that in prostate hyperplasia patients (*P* < 0.05). The analysis results indicated that the f/t ratio and the relative expression of miRNA-222 and miRNA-1248 were independent influences of prostate cancer (*P* < 0.05), in which overexpression of miRNA-222 decreased the proliferative, invasive, and metastatic abilities of PC3 cells and enhanced the level of apoptosis of cancer cells.

**Conclusions:**

Although there was no significant change in the overall incidence of prostate cancer in this study, significant changes occurred in the incidence of prostate cancer with different characteristics. In addition, the nomogram prediction model of prostate cancer-specific survival rate constructed based on four factors has a high reference value, which helps physicians to correctly assess the patient-specific survival rate and provides a reference basis for patient diagnosis and prognosis evaluation.

**Supplementary Information:**

The online version contains supplementary material available at 10.1186/s12957-023-03151-1.

## Introduction

Prostate cancer (PCa) is one of the leading causes of cancer-related deaths worldwide and is currently the second most common malignancy in men worldwide [1, 2]. Prostate cancer accounts for about one-third of malignant tumors in men, and 80–90% of patients have progressed to mid to late stage by the time they are first diagnosed with prostate cancer [3], mostly in older men. The elderly population not only has a high incidence of prostate cancer, but also has a high degree of malignancy and a low survival rate [4, 5]. In order to reduce the risk of prostate cancer and improve the quality of patient survival, patients with prostate cancer need to be detected and treated at an early stage. Therefore, finding indicators that can screen for prostate cancer at an early stage and treatments that can delay and stop the onset and progression of prostate cancer are the focus of current prostate cancer research [6].

Many recent studies have shown that micro RNA (miRNA), an important molecule proven to be closely related to tumorigenesis and development, has potential diagnostic, therapeutic, and prognostic values for tumors and is currently the focus of oncology research [7]. It has been found that there is some variation in the expression levels of some miRNAs in the plasma or serum of prostate cancer patients, and the miRNAs in plasma or serum are in a stable form due to microbubble protection from most RNA degradation agents, which is the advantage that miRNA can be a new detection indicator for prostate cancer [8, 9]. Therefore, it can be concluded that miRNAs in the peripheral circulation of prostate cancer patients have stability, specificity, and sensitivity in the diagnosis of prostate cancer, and miRNAs in the circulating peripheral circulation of patients can be used as biomarkers for the diagnosis of prostate cancer.

In summary, miRNAs are of great value in prostate cancer diagnosis, treatment, and nausea progression. In this study, we screened differentially expressed miRNAs in prostate cancer tissues and prostate z hyperplasia tissues by miRNA microarrays to analyze the potential of miRNAs in prostate cancer diagnosis, treatment, and tumor nausea progression.

## Research content and methods

### Study subjects and clinical samples

#### Source of tissue samples

Prostate tissue samples were collected from 37 patients with prostate cancer and 41 patients with prostate hyperplasia diagnosed by pathology in the Department of Urology of the First Affiliated Hospital of Xinjiang Medical University from October 2021 to July 2022, and the samples were stored in a refrigerator at − 80℃. The collected tissue samples were used for miRNAs microarray and qRT-PCR assay to verify the expression of miRNAs.

#### Source of blood samples

Blood samples were collected from 52 patients with prostate cancer and 58 patients with prostate hyperplasia diagnosed by pathology in the Department of Urology of the First Affiliated Hospital of Xinjiang Medical University from November 2021 to May 2022, and the samples were stored in a refrigerator at − 80℃. The collected blood samples were used for miRNAs microarray and qRT-PCR assay to verify the expression of miRNAs.

#### General clinical data

Nineteen general clinical data were collected on the above patients, including age, education, domicile, marital status, alcohol consumption, smoking, hypertension, body mass index (BMI), diabetes, PSA, free prostate antigen/total prostate antigen (f/t) ratio, triglycerides, potassium, calcium, low-density lipoprotein, testosterone, prostate volume, total cholesterol, and alkaline phosphatase.

Inclusion criteria include patients who underwent prostate puncture biopsy for the first time, patients with a clear diagnosis by pathological tissue biopsy, patients with complete clinical information, and patients who signed an informed consent form agreeing to participate in this study.

Exclusion criteria include patients who were not diagnosed with prostate cancer for the first time, patients with other malignant tumors in combination, patients who had received oncological treatment or treatment for prostate-related diseases, and patients who did not want to participate in this study.

### Experimental methods

#### MicroRNAs microarray assay

Tissues of 4 patients with prostate cancer and 4 patients with prostate hyperplasia were randomly selected. Extraction of total RNA, detection of RNA quality, library construction, removal of rRNA, RNA fragmentation, reverse transcription, amplification, and up-sequencing.

#### Detection of miRNA expression in tissue and blood samples by fluorescence real-time quantitative PCR

Grinding of tissue samples using a tissue grinder (Extraction of lymphocytes from blood samples). Extraction of total RNA from tissue samples and lymphocytes by TRIzol method for RNA concentration and purity testing. The miRNA reverse transcription reagent was used to configure the reverse transcription reaction solution and placed in a thermocycler with temperature and time settings: a warm bath at 37℃ for 60 min, followed by heating at 85℃ for 5 min to inactivate the enzyme and reverse transcribe the RNA into cDNA. The miRNA fluorescent quantitative PCR kit (dye method) reagents were used, and after configuring the PCR amplification reaction solution, the PCR amplification program (Table 1) was designed and amplified in a fluorescent quantitative qRT-PCR instrument. Patients with prostate enlargement were used as controls, and the relative expression of miRNAs was calculated using 2^−∆∆Ct^ (Table 2).

**Table 1:** miRNA real-time fluorescence quantification reaction conditions

Steps	Temperature	Time	Number of cycles
Predegeneration	95℃	30 s	
Degeneration	95℃	5 s	40 cycles
Annealing/Extension	60℃	30 s	

The melting curve analysis should be performed according to the recommended procedure of the fluorescence PCR instrument used

**Table 2:** PCR primer sequences

Genes		Primer sequences
U6	F	CGTCAACACTTGCTGGT
	R	CTCGCTTCGGCAGCACA
miRNA-144	F	TACAGTATAGATGATGTACT
miRNA-222	F	AGCTACATCTGGCTACTGGGT
miRNA-1248	F	ACCTTCTTGTATAAGCACTGTGCTAAA
miRNA-3651	F	CATAGCCCGGTCGCTGGTACATGA

### Cellular experiments

#### Cell source

Human prostate cancer PC-3 cells and human normal prostate wpmy-1 cells, purchased from Wuhan Pronosai Biological Company.

#### Cell culture

The complete medium for wpmy-1 cells contains 10% fetal bovine serum, DMEM high sugar basal medium, 1% penicillin, and streptomycin mixture. The complete medium for PC-3 cells contains 10% fetal bovine serum, DME/F-12 basal medium, 1% penicillin, and streptomycin mixture. Incubation was performed in an incubator at 37℃ with 5% CO_2_. The medium can be changed after 48–72 h of incubation in the incubator, the cells can be passaged when the cell fusion rate reaches 80–100%. The cells are frozen using 10% DMSO + 90% fetal bovine serum for cell freezing.

#### Detection of miRNA expression in cells

Cells with 80–100% fusion rate were washed 1–2 times with PBS and centrifuged after cell digestion with trypsin. TRIzol was added to the precipitated cells and transferred to a 1.5-ml centrifuge tube and blown and mixed. The subsequent RNA extraction, cDNA synthesis, and qRT-PCR steps were performed for tissue and blood samples, using wpmy-1 cells and control PC-3 cells as control groups. The relative expression of miRNA was calculated in the same way as for tissue and blood samples.

#### Construction of prostate cancer transfected cells to interfere with miRNA expression

Design and synthesis of siRNAs. The miRNA-222-mimics and negative controls were designed and synthesized by Jima Genetics, Shanghai, China (Table 3).

**Table 3:** Transfection sequence of siRNA

Gene names	Sequence	
miRNA-222-mimics	Sense	AGCUACAUCUGGCUACUGGGU
	Antisense	CCAGUAGCCAGAUGUAGCUUU
NC	Sense	UUCUCCGAACGUGUCACGUTT
	Antisense	ACGUGACACGUUCGGAGAATT

Cells were transfected in three groups: miRNA-222-mimics group, NC group, and control group. miRNA-222-mimics is the group in which miRNA-222 is overexpressed in cells. NC group is used to exclude cells from the effect of transfection reagent. The control group is the same batch of cells as miRNA-222-mimics and the NC group without transfection. These three groups were cultured simultaneously. Since the medium and reagents used in the transfection process did not add double antibodies (penicillin and streptomycin mixture), an aseptic operation was required to avoid contamination of the cells. Before transfection, cells with 80–100% fusion were digested and inoculated in six-well plates with antibody-free medium, and when the fusion of cells in the six-well plates reached 50–70%, transfection could be performed:

1. Dilute miRNA-222-mimics and NC with DEPC water and adjust to a concentration of 10 µM.
2. Add 12 µl of transfection reagent to 240 µl of DME/F-12 basal medium, add 6 µl of diluted miRNA-222-mimics and NC to 120 µl of DME/F-12 basal medium respectively, and leave at room temperature for 5 min.
3. Add 126 µl of miRNA-222-mimics and NC dilutions respectively from transfection reagent dilutions and mix by blowing, leave at room temperature for 20–25 min. Wash the cells in six-well plates with PBS in advance and add 2 ml of antibody-free medium to each well.
4. Add 252 µl of miRNA-222-mimics and NC mixture to each well in a six-well plate and incubate in an incubator for 48–72 h for subsequent experiments after successful transfection.

#### CCK8 assay for cell proliferation

1. The 3 groups of successfully transfected cells were washed with PBS, and the cells were collected and counted after digestion.
2. 96-well plates are added with media containing 6000 cells at 100 µl per well. For each group, 18 replicate wells were set up, and a negative control well with only medium was designed.
3. After 24, 48, and 72 h of cell walling, 10 µl of CCK-8 was added into 6 replicate wells of each group of cells for 2 h, the OD of each well at 450 nm was measured by Microplate Reader, the average OD of each replicate well was taken, the average OD of the negative control group was subtracted from the average OD of each group, the proliferation inhibition rate of miRNA-222-mimics group and NC group was calculated according to the CCK-8 reagent instructions.

#### Scratching experiment

1. Before transfection, use a marker to draw 5 marker lines on the back of the six-well plate before transfection. After successful transfection, when the fusion of transfected cells reaches 80–100%, use a 200-µl pipette to scratch in each well of cells, and scratch perpendicular to the marker lines.
2. After scratching, wash several times with PBS, add low-serum medium, and record each scratch area with microscope photos at 0, 24, and 48 h after scratching, and take photos of the same scratch area at different times and record the location.
3. The images were analyzed using Image J software, and when calculating the relative area of scratches at 24 and 48 h, the scratch area at 0 h for each group was used as a reference value to evaluate the migration ability of each group of cells.

#### Transwell experiments

1. Before starting the experiment, pre-chill the centrifuge tubes, pipettes, and tips on ice to avoid solidification of the Matrigel matrix gel at room temperature.
2. Dilute Matrigel base gel with DME/F-12 basal medium at a ratio of 1:8 and add 60 µl to the upper layer of Transwell (be careful to avoid foaming when adding Matrigel base gel), leave in the incubator for 1–2 h to form a film and discard any excess liquid that has not solidified.
3. The 3 groups of successfully transfected cells were washed using PBS, digested and centrifuged, and then suspended in serum-free medium and counted. Two hundred microliters of serum-free medium containing 30,000 cells was inoculated in the upper layer of Transwell, and 600 µl of medium containing 10% serum was added to the lower layer and placed in the incubator for 36 h.
4. After washing the removed Transwell with PBS, aspirate the excess liquid in the upper chamber, add 600 µl of 4% paraformaldehyde for fixation to the wells in the upper chamber, and fix for 20–30 min.
5. After fixation, the upper chamber of Transwell was washed several times with PBS, and the upper chamber was transferred to the wells stained with 600 µl of 0.1% crystal violet staining solution added in advance for 5–10 min.
6. After staining, the Transwell upper chamber was washed several times with PBS, and the background color was removed and air-dried for 10 min. After wiping the unsuccessful cells on the bottom surface of the upper chamber with a wet cotton swab, five fields of view were randomly selected and photographed under the microscope, and the successfully invaded cells were counted using Image J software.

#### Flow cytometry

1. After the supernatant of each group of cells and the digested cells were collected, they were centrifuged together, the supernatant was discarded, and the cell precipitate was washed several times with PBS.
2. Add 100 µl of 1 × binding buffer to each group of cell sediment and transfer to a 1.5-ml centrifuge tube, add 5 µl of PI staining solution and stain for 3 min, then add 5 µl of lAnnexinV-FITC staining solution for co-staining for 5 min, and finally, add 400 µl of 1 × binding buffer to each group of cells to terminate staining (all experimental steps after the start of staining need to be operated under light-proof conditions).
3. After filtering each group of cells into a single dispersed state with a filter membrane, apoptosis detection was performed using flow cytometry.
4. The percentages of the Q1, Q2, Q3, and Q4 will appear in the test results. The percentage of the Q1 area represents the percentage of mechanically damaged cells caused by operation errors in the total cell count. The percentage of the Q2 area represents the percentage of necrotic or late apoptotic cells in the total cell count. The percentage of the Q3 area represents the percentage of early apoptotic cells in the total cell count. The percentage of the Q4 area represents the percentage of normal cells in the total cell count.

#### Statistical method

Graphpad Prism 5 and SPSS 25.0 software were used for graphing and analysis. The measurement data obeying normal distribution were expressed as mean ± standard deviation ($\left(\overline x\;\pm\;s\right)$ and those not obeying normal distribution were expressed as quartiles *M*(*P*_25_, *P*_75_), and the count data were described as percentages (%). For single-factor analysis, data obeying normal distribution were analyzed by *t*-test, data not obeying normal distribution were analyzed by rank sum test, and count data were analyzed by chi-square test. For multi-factor analysis, unconditional logistic regression was used for the analysis. The test level was 0.05.

## Results

### Prostate tissue miRNA microarray results

To explore the expression of miRNAs in prostate cancer and prostate hyperplasia tissues, four cases of prostate cancer tissues (Ca2, Ca4, Ca5, Ca14) and four cases of prostate hyperplasia tissues (N7, N11, N15, N16) were selected, all of which were obtained from puncture samples from the Department of Urology of the First Affiliated Hospital of Xinjiang Medical University from October 2021 to January 2022.

#### Sequencing data quality

The percentages of pure sequences in the original sequences of the four prostate cancer tissue samples were Ca2 96.69%, Ca4 96.27%, Ca5 96.40%, and Ca14 93.62%, all of which were greater than 90%. The percentages of pure sequences in the original sequences of the four prostate hyperplasia tissue samples were N7 96.80%, N11 97.23%, N15 95.72%, and N16 96.68%, all of which were greater than 90%. The pure sequence ratio and Q30 of the eight tissue samples were greater than 90%, which can indicate the good quality of sequencing data (Table 4).

**Table 4:** Original and post-quality control sequence information in prostate cancer tissue and prostate cancer hyperplasia tissue

Sample name	Number of original sequences (*n*)	Number of pure sequences (*n*)	Pure sequence ratio (%)	Q30 (%)
Ca2	12,231,830	11,827,333	96.69%	94.64%
Ca4	15,529,734	14,950,214	96.27%	97.08%
Ca5	13,979,225	13,475,826	96.40%	96.33%
Ca14	11,507,052	10,772,859	93.62%	96.09%
N7	14,999,205	14,448,964	96.33%	96.80%
N11	18,063,727	17,452,607	96.62%	97.23%
N15	14,306,038	13,662,588	95.50%	95.72%
N16	13,362,300	12,693,394	94.99%	96.68%

#### Sequence length distribution characteristics

In the data analysis, the sequences with length < 17 bp or > 35 bp were removed. Four cases of prostate cancer tissues with Small RNA pure sequences were mainly distributed in the length of 20 ~ 24 bp, mainly concentrated in 22 bp, and the percentage of pure sequences with length in 22 bp were Ca2 41.87%, Ca4 31.75%, Ca5 30.72%, and Ca14 41.97%. Four cases of prostate cancer tissues with small RNA pure sequences were mainly distributed in the length of 20 ~ 24 bp, mainly concentrated in 22 bp, and the percentage of pure sequences with length in 22 bp were Ca2 41.87%, Ca4 31.75%, Ca5 30.72%, and Ca14 41.97%. The percentages of pure sequences with 22 bp were N7 38.61%, N11 31.76%, N15 32.43%, and N16 38.42%, respectively. Compared with prostate cancer tissue samples, the distribution of pure sequence lengths in prostate hyperplasia tissue samples was more uniform.

#### Taxonomic annotation of small RNA sequences

Using blastn software, we compared the pure sequences with Rfam (version 10.0) database and extracted the results with *p*-value less than or equal to 0.01. The number of successful sequences among the pure sequences in the four prostate cancer tissues was Ca2 78.55%, Ca4 74.38%, Ca5 70.70%, and Ca14 72.92%, among which the percentages of miRNAs in pure sequences in the four prostate hyperplasia tissues were N7 83.97%, N11 74.68%, N15 67.80%, and N16 72.67%, of which miRNAs were N7 77.12%, N11 74.68%, N15 67.80%, and N16 72.67%, respectively. N7 77.12%, N11 62.49%, N15 55.76%, and N16 61.06%, respectively, are shown in eTable 1.

#### Expression of total miRNA

**Fig. 1:**
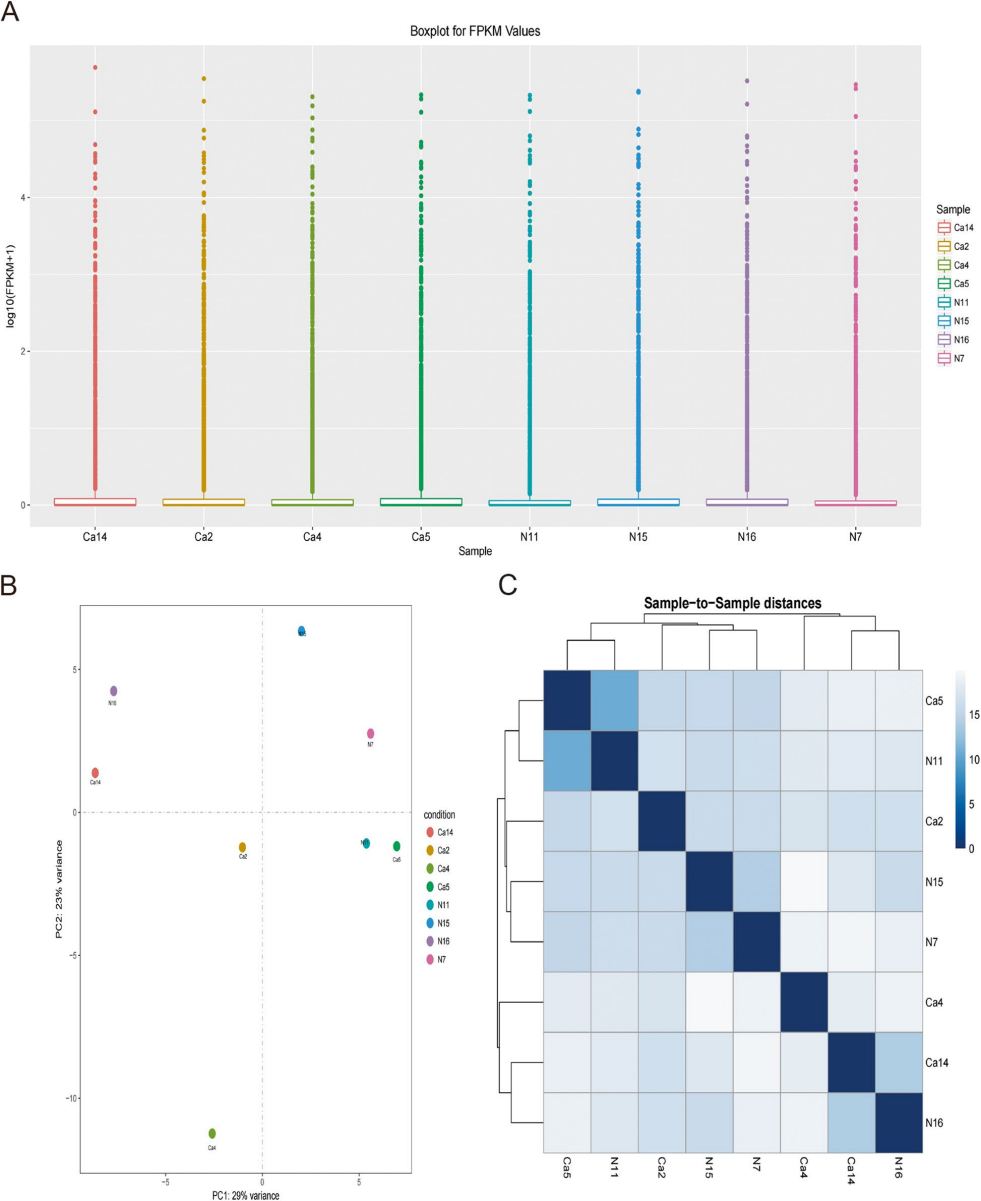
**A** Box plot of miRNA expression levels in prostate cancer tissue and prostate cancer hyperplasia tissue. **B** PCA images of prostate cancer tissue and prostate cancer hyperplasia tissue. **C** Cluster analysis graph between samples

In small RNA sequencing analysis, we can estimate the miRNA expression levels by the counts of newly predicted miRNA sequences in different samples (Fig. 1A). miRNA expression levels of samples in the cancer and hyperplasia groups were not significantly different, and the expression was mainly concentrated in 1 ~ 3. The similarity between different samples can be explained by PCA and the PCA distance between samples or sample clustering. The closer the distance, the higher the similarity between samples. For each group of samples distributed in different regions of two-dimensional space, samples in the same group were relatively concentrated in spatial distribution. In this study, PCA could be seen that the prostate cancer group was concentrated in the lower part of the two-dimensional space, and the prostate hyperplasia group was concentrated in the upper part of the two-dimensional space (Fig. 1B). When examining the similarity between different samples, the distance between different samples was calculated by the clustering method, and in this study, the clustering method could see that the distance between the samples of the prostate cancer group was more similar (Fig. 1C).

### Identification of differential miRNAs

**Fig. 2:**
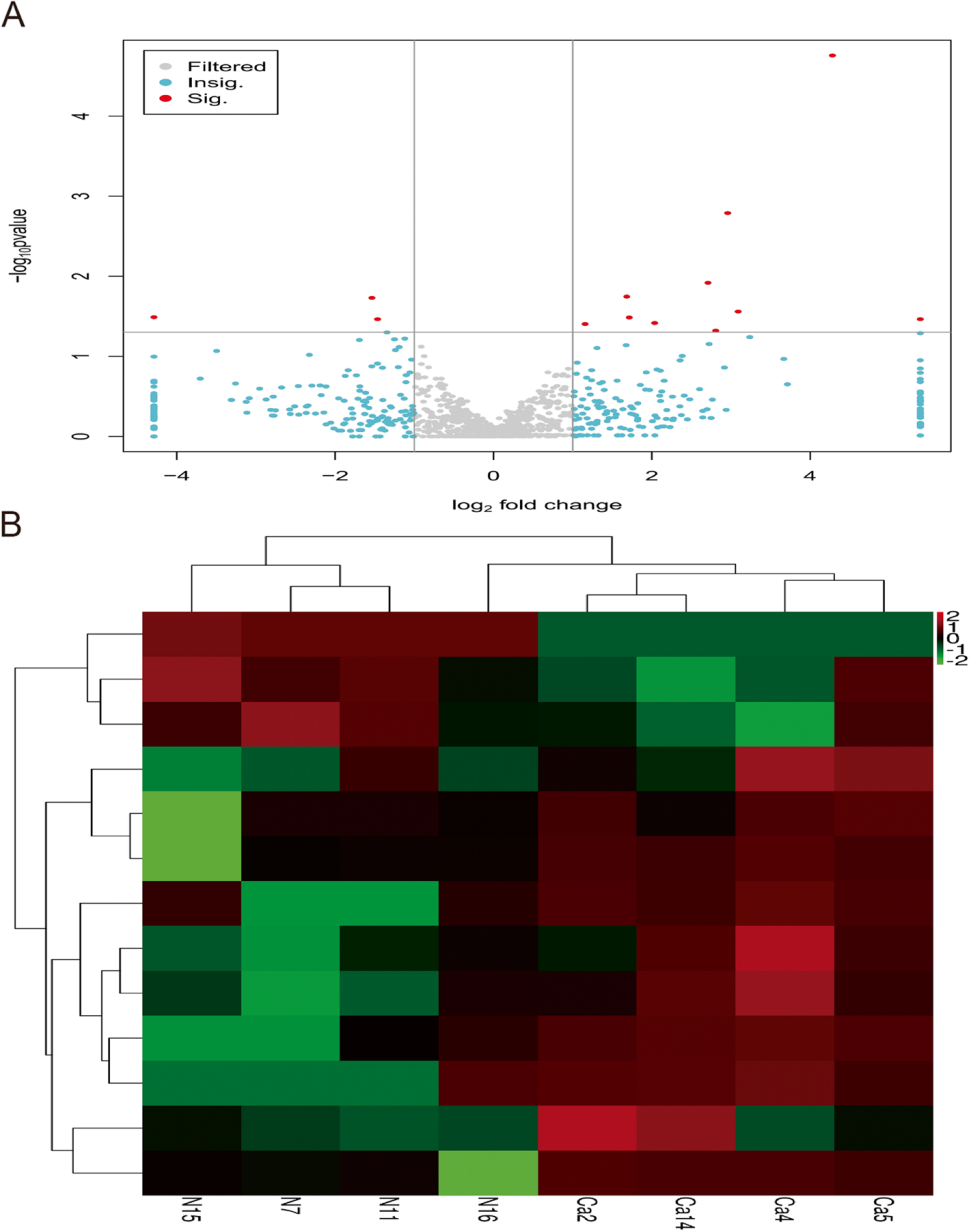
**A** Volcano plot of miRNA differential expression profiles in prostate cancer tissue and prostate cancer hyperplasia tissue. **B** Heat map of miRNA differential expression profiles in prostate cancer tissue and prostate cancer hyperplasia tissue

A negative binomial distribution test was used to test the significance of differences in sequence numbers. Base mean values were used to estimate miRNA expression, and miRNAs with *P* < 0.05 and with more than twofold change were screened. A total of 13 miRNAs were screened for differential expression in prostate cancer and prostate hyperplasia tissues, of which 10 were upregulated in expression miRNAs (hsa-miRNA-1248, hsa-miRNA-144-3p, hsa-miRNA-182-3p, hsa-miRNA-3609, hsa-miRNA-3651, hsa-miRNA-449c-5p, and four unknown miRNAs were expressed significantly higher in prostate cancer tissues compared with prostate hyperplasia tissues), and three miRNAs with down-regulated expression (hsa-miRNA-1299, hsa-miRNA-205-3p, and hsa-miRNA-222-3p were significantly lower in prostate cancer tissues compared with prostate hyperplasia tissues) (Table 5). The gray dots in the volcano plot represent miRNAs that were not differentially expressed in prostate cancer and prostate hyperplasia tissues, and the red and green dots represent miRNAs that were differentially expressed in prostate cancer and prostate hyperplasia tissues, with the red dots indicating miRNAs that were screened out with *P* < 0.05 and more than twofold change (Fig. 2A). In order to visualize the miRNAs differentially expressed in prostate cancer and prostate hyperplasia tissues, cluster analysis was performed on the data, and the clustering analysis plot with green dots indicating low expression and red dots indicating high expression showed that the expression levels of the screened miRNAs in prostate cancer and prostate hyperplasia tissues were different (Fig. 2B).

**Table 5:** Differential expression of miRNAs in prostate cancer tissues and prostate cancer hyperplasia tissues

miRNA	Expression of miRNA after standardization		Fold change	*P*	Up/down
	Prostatic hyperplasia	Prostate cancer			
chr15_14511	1.393	10.815	7.766	0.002	Up
chr20_17438_star	0.986	19.151	19.426	0.000	Up
chr20_17674	95.176	312.417	3.283	0.033	Up
chr4_5055	1.746	73.182	41.914	0.034	Up
hsa-miR-1248	136.837	305.092	2.230	0.039	Up
hsa-miR-1299	150.420	51.888	0.345	0.019	Down
hsa-miR-144-3p	2.542	10.421	4.099	0.038	Up
hsa-miR-182-3p	1.519	9.926	6.534	0.012	Up
hsa-miR-205-3p	3.165	0.000	0.000	0.032	Down
hsa-miR-222-3p	601.264	217.983	0.363	0.034	Down
hsa-miR-3609	0.866	6.053	6.993	0.048	Up
hsa-miR-3651	6.945	22.302	3.211	0.018	Up
hsa-miR-449c-5p	0.537	4.576	8.514	0.028	Up

#### Functional analysis of differential miRNA target genes

##### Functional analysis of differential miRNA target genes

**Fig. 3:**
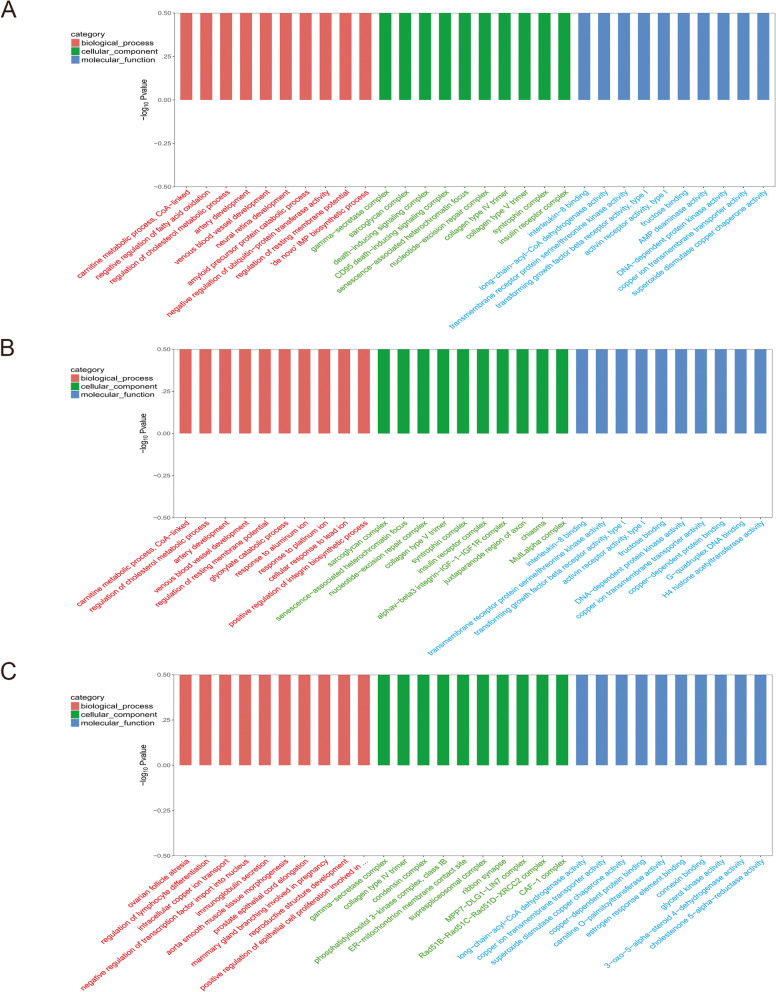
Results of GO analysis of miRNA target genes differentially expressed in prostate cancer tissue and prostate hyperplasia tissue. **A** All miRNAs. **B** Highly expressed miRNA. **C** Lowly expressed miRNA

GO functional significance enrichment analysis was performed on the target genes of 13 all miRNAs differentially expressed (Fig. 3A), 10 miRNAs highly expressed in prostate cancer (Fig. 3B), and 3 miRNAs low expressed in prostate cancer (Fig. 3C), respectively. The results of GO functional significance enrichment analysis of 13 miRNAs differentially expressed in the cellular processes indicated that these miRNAs were enriched in carnitine metabolic process (CoA-related), negative regulation of fatty acid oxidation, regulation of cholesterol metabolic process, arterial development, venous vascular development, neuroretinal development, amyloid precursor protein catabolic process, negative regulation of ubiquitin-protein transferase activity, regulation of resting membrane potential, “de novo.” The molecular functions include interleukin-8 binding, long-chain acyl-coenzyme A dehydrogenase activity, transmembrane receptor protein serine/threonine kinase activity, transforming growth factor beta receptor activity (type I), activin receptor activity (type I), fructose binding, AMP deaminase activity, DNA-dependent protein kinase activity, copper ion transmembrane transporter protein activity, and superoxide dismutase copper chaperone activity. The cellular composition includes γ-secretase complex, myoglycan complex, death-inducing signaling complex, CD95 death-inducing signaling complex, senescence-associated heterochromatin foci, nucleotide excision repair complex, type IV collagen trimer, type V collagen trimer, synthetic nutrient complex, and insulin receptor complex.

##### KEGG pathway analysis

**Fig. 4:**
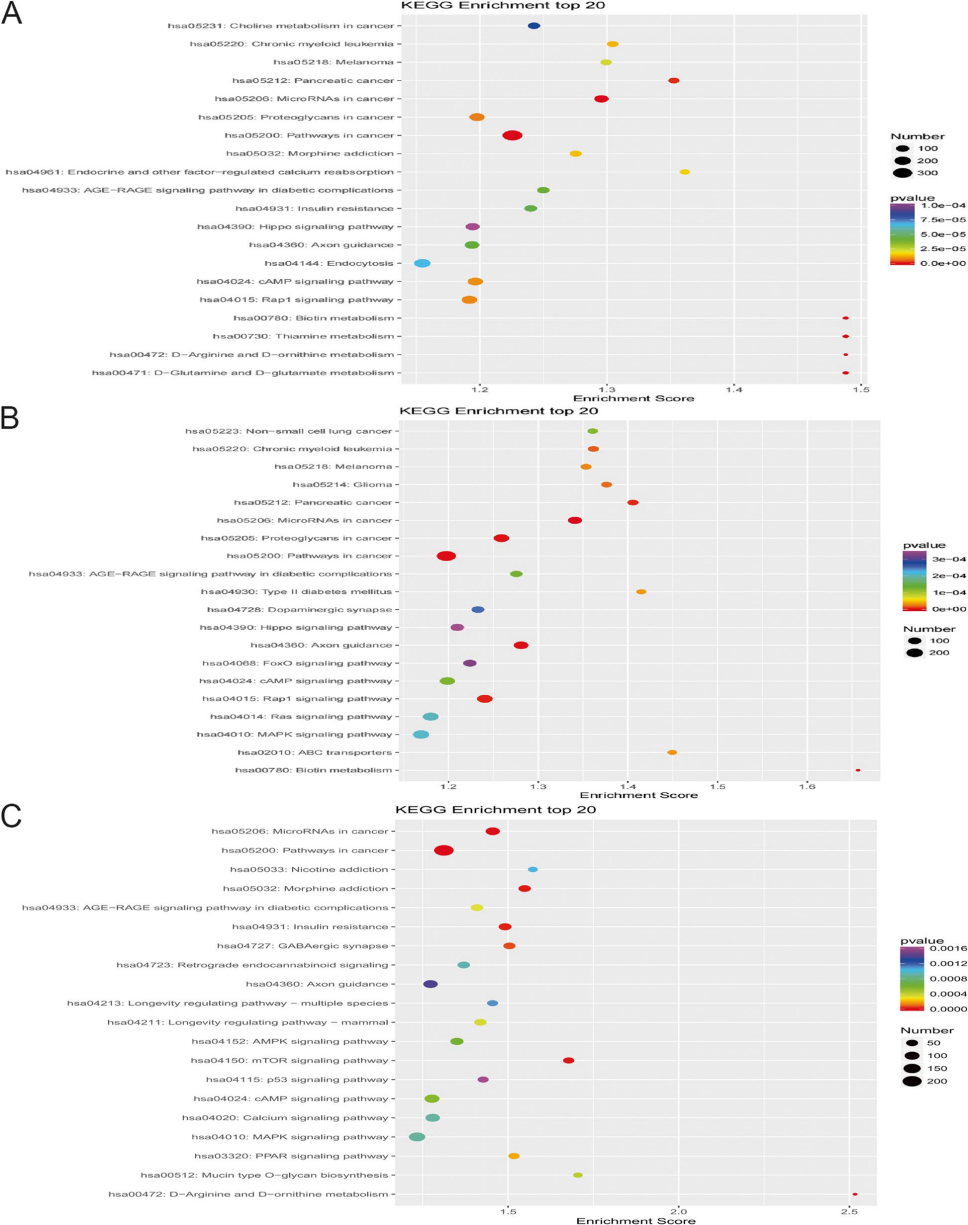
Results of KEGG analysis of miRNA target genes differentially expressed in prostate cancer tissue and prostate hyperplasia tissue. **A** All miRNAs. **B** Highly expressed miRNA. **C** Lowly expressed miRNA

After obtaining the differentially expressed miRNAs between the four prostate cancer tissues and the four prostate hyperplasia tissues, the target genes were predicted by KEGG enrichment analysis for the 13 differentially expressed all miRNAs (Fig. 4A), the 10 miRNAs that were highly expressed in prostate cancer (Fig. 4B) and the three miRNAs that were lowly expressed in prostate cancer (Fig. 4C), respectively. The results of KEGG enrichment analysis of 13 differentially expressed all miRNAs indicated that the target genes of these miRNAs were mainly involved in the tumor-associated protein pathway, miRNAs-associated protein pathway in tumor, cAMP signaling pathway, and Rap1 signaling pathway.

### Results of miRNA relative expression in tissue samples

By reviewing the literature, we selected four known miRNAs that have rarely been studied in prostate cancer. Three miRNAs with up-regulated levels were hsa-miRNA-144, hsa-miRNA-1248, and hsa-miRNA-3651, and one miRNA with down-regulated levels was hsa-miRNA-222 for the next study.

Thirty-three patients with prostate cancer, with a mean age of (69.30 ± 7.72) years, and 37 patients with prostate hyperplasia group, with a mean age of (66.46 ± 7.77) years, were included in this study. The results showed that the relative expression of miRNA-144, miRNA-1248, and miRNA-3651 was not statistically significant between the prostate cancer and prostate hyperplasia groups. miRNA-222 was statistically significant between the prostate cancer and prostate hyperplasia groups, and miRNA-222 was significantly low expressed in the tissues of prostate cancer patients (*P* < 0.05) (Table 6).

**Table 6:** miRNA expression in tissues of patients with prostate hyperplasia and prostate cancer

miRNA	Prostate cancer	Prostate hyperplasia	*Z*	*P*
miRNA-144	1.91 (0.65,3.19)	2.30 (0.52,5.80)	− 0.747	0.455
miRNA-222	0.65 (0.52,1.34)	1.28 (0.85,1.70)	− 2.700	0.007
miRNA-1248	1.17 (0.54,1.83)	1.06 (0.43,2.01)	− 0.076	0.939
miRNA-3651	0.84 (0.64,1.25)	1.10 (0.69,1.25)	− 1.018	0.309

### Results of miRNA relative expression in blood samples

Fifty-two patients with prostate cancer and 58 patients with prostate hyperplasia were included in this study. miRNA-144 had no statistically significant relative expression in the blood of patients with prostate cancer and prostate hyperplasia. miRNA-222, miRNA-1248, and miRNA-3651 were significantly low expressed in the blood of patients with prostate cancer (*P* < 0.05) (Table 7).

**Table 7:** miRNA expression in the blood of patients with prostate cancer and prostate hyperplasia

miRNA	Prostate Cancer	Prostate hyperplasia	*Z*	*P*
miRNA-144	0.90 (0.48,1.36)	1.33 (0.63,1.55)	− 1.701	0.089
miRNA-222	0.56 (0.43,0.63)	0.95 (0.74,1.31)	− 8.263	< 0.001
miRNA-1248	0.90 (0.77,0.98)	0.97 (0.87,1.19)	− 2.994	0.003
miRNA-3651	0.87 (0.79,0.98)	1.00 (0.86,1.15)	− 3.209	0.001

### Blood miRNA expression in different clinical T-stages, Gleason scores and metastatic conditions

Comparing the expression of miRNA-144, miRNA-222, miRNA-1248, and miRNA-3651 in the blood of patients with different clinical T-stage, Gleason score, and metastatic status. The results showed that the expression of miRNA-144, miRNA-222, miRNA-1248, and miRNA-3651 in the blood of patients with different clinical T-stage, Gleason score, and metastatic status was not statistically different (*P* > 0.05) (Table 8).

**Table 8:** miRNA expression in the blood of patients with different clinical T-stage, Gleason score, and metastatic status

Variables	miRNA-144	miRNA-222	miRNA-1248	miRNA-3651
Clinical T-stage				
≤ 2	0.73 (0.40,1.06)	0.54 (0.43,0.59)	0.84 (0.75,0.96)	0.87 (0.83,1.00)
> 2	1.00 (0.65,2.31)	0.56(0.35,0.62)	0.91 (0.78,1.02)	0.80 (0.63,0.95)
*Z*	− 1.665	− 0.333	− 0.916	− 1.498
*P*	0.096	0.739	0.360	0.134
Gleason score				
≤ 7	0.91 (0.44,1.29)	0.54 (0.42,0.57)	0.79 (0.88,0.96)	0.88 ± 0.06
> 7	0.80 (0.48,1.36)	0.56 (0.45,0.63)	0.89 (0.77,0.98)	0.93 ± 0.29
*Z*/*t*	− 0.547	− 1.300	− 0.684	− 0.650
*P*	0.584	0.194	0.494	0.518
metastatic status				
No	0.90 (0.33,1.34)	0.54 (0.42,0.65)	0.82 (0.71,0.95)	0.86 (0.79,0.95)
Yes	0.80 (0.68,1.35)	0.56 (0.42,0.58)	0.96 (0.82,0.99)	0.87 (0.76,1.06)
*Z*	− 0.423	− 0.962	− 1.347	− 0.038
*P*	0.672	0.336	0.178	0.969

### Prostate cancer risk factors

#### Univariate analysis of risk factors for prostate cancer

General clinical data of 52 patients with prostate cancer and 58 patients with prostate hyperplasia were changed to categorical data for univariate analysis of prostate cancer. Among them, the PSA value was divided into dichotomous variables by 10 ng/ml, age by 65 years, and BMI by 24 kg/m2. f/t ratio, alkaline phosphatase, prostate volume, testosterone, miRNA-144 relative expression, miRNA-222 relative expression, miRNA-1248 relative expression, and miRNA-3651 relative expression were divided into dichotomous variables by the median. Total cholesterol was divided into dichotomous variables with the mean as the cut-off. Low-density lipoprotein, blood calcium, blood potassium, and triglycerides were divided into two groups with the lower limit of the normal range of each index as the boundary value.

The analysis showed that there were no statistically significant differences (*P* > 0.05) when comparing the education level, household registration, alcohol consumption, marital status, smoking, hypertension, diabetes, BMI, LDL, blood calcium, blood potassium, triglycerides, testosterone, prostate volume, total cholesterol, alkaline phosphatase, and miRNA-144 between patients with prostate hyperplasia and prostate cancer. Age, PSA, f/t ratio, miRNA-222, miRNA-1248, and miRNA-3651 relative expression were statistically different (*P* < 0.05) (Table 9).

**Table 9:** Univariate analysis of risk factors for prostate cancer

Variables	Prostate hyperplasia *n* (%)	Prostate cancer *n* (%)	*χ*^2^	*P*
Age			6.298	0.012
≤ 65	24 (41.4)	10 (19.2)		
> 65	34 (58.6)	42 (80.8)		
Education level			2.713	0.100
Junior high school and below	38 (65.5)	26 (50.0)		
High school and above	20 (34.5)	26 (50.0)		
Place of residence			0.717	0.397
Urban	48 (82.8)	46 (88.5)		
Rural	10 (17.2)	6 (11.5)		
Marital status			0.495	0.482
Married	54 (93.1)	50 (96.2)		
Divorced or widowed	4 (6.9)	2 (3.8)		
Smoking			0.073	0.787
No	48 (82.8)	42 (80.8)		
Yes	10 (17.2)	10 (19.2)		
Drinking			2.661	0.103
No	56 (96.6)	46 (88.5)		
Yes	2 (3.4)	6 (11.5)		
High blood pressure			1.624	0.203
No	36 (62.1)	26 (50.0)		
Yes	22 (37.9)	26 (50.0)		
Diabetes			3.248	0.072
No	52 (89.7)	40 (76.9)		
Yes	6 (10.3)	12 (23.1)		
BMI			0.340	0.560
≤ 24	28 (48.3)	28 (53.8)		
> 24	30 (51.7)	24 (46.2)		
PSA			14.237	< 0.001
≤ 10	34 (58.6)	12 (23.1)		
> 10	24 (41.4)	40 (76.9)		
f/t ratio			6.639	0.010
≤ 0.18	26 (44.8)	36 (69.2)		
> 0.18	32 (55.2)	16 (30.8)		
Triglycerides			0.033	0.857
≤ 1.7	28 (48.3)	26 (50.0)		
> 1.7	30 (51.7)	26 (50.0)		
Blood potassium			3.216	0.073
≤ 3.5	20 (34.5)	10 (19.2)		
> 3.5	38 (65.5)	42 (80.8)		
Blood Calcium			0.019	0.889
≤ 2.25	32 (55.2)	28 (53.8)		
> 2.25	26 (44.8)	24 (46.2)		
Low-density lipoprotein			0.026	0.873
≤ 3.4	54 (93.1)	48 (92.3)		
> 3.4	4 (6.9)	4 (7.7)		
Testosterone			0.019	0.889
≤ 17.08	32 (55.2)	28 (53.8)		
> 17.08	26 (44.8)	24 (46.2)		
Prostate volume			0.294	0.587
≤ 94.16	26 (44.8)	26 (50.0)		
> 94.16	32 (55.2)	26 (50.0)		
Alkaline phosphatase			0.033	0.857
≤ 66.41	28 (48.3)	26 (50.0)		
> 66.41	30 (51.7)	26 (50.0)		
Total cholesterol			0.340	0.560
≤ 4.11	30 (51.7)	24 (46.2)		
> 4.11	28 (48.3)	28 (53.8)		
miRNA-144			0.892	0.345
≤ 0.92	26 (44.8)	28 (53.8)		
> 0.92	32 (55.2)	24 (46.2)		
miRNA-222			61.167	< 0.001
≤ 0.69	8 (13.8)	46 (88.5)		
> 0.69	50 (86.2)	6 (11.5)		
miRNA-1248			4.458	0.035
≤ 0.94	24 (41.4)	32 (61.5)		
> 0.94	34 (58.6)	20 (38.5)		
miRNA-3651			8.269	0.004
≤ 0.93	22 (37.9)	34 (65.4)		
> 0.93	36 (62.1)	18 (34.6)		

#### Unconditional logistic regression analysis

The presence or absence of prostate cancer was used as the dependent variable, and the factors with statistical differences in univariate analysis, age, PSA, f/t ratio, miRNA-222, miRNA-1248, and miRNA-3651 were used as independent variables (Table 10). The stepwise backward method was used for the selection and exclusion of independent variables, setting α exclusion = 0.10 and α entry = 0.05. The results of multivariate analysis showed that a total of three indicators/variables, such as f/t ratio, miRNA-222, and miRNA-1248, were retained into the regression equation and were independent influencing factors for prostate cancer (*P* < 0.05), and f/t ratio > 0.18, miRNA-222 relative expression > 0.69 and miRNA-1248 relative expression > 0.94 were protective factors for prostate cancer (Table 11). The ROC curve area was 0.941 with a 95% confidence interval of 0.900 to 0.982 when the three indicators/variables of f/t ratio, miRNA-222, and miRNA-1248 were used as reference values to differentiate prostate hyperplasia from prostate cancer.

**Table 10:** Logistic regression variable assignment

Factors	Assignments	
Age	≤ 65:1	> 65:2
PSA	≤ 10:1	> 10:2
f/t ratio	≤ 0.18:1	> 0.18:2
miRNA-222	≤ 0.69:1	> 0.69:2
miRNA-1248	≤ 0.94:1	> 0.94:2
miRNA-3651	≤ 0.93:1	> 0.93:2

**Table 11:** Multi-factor logistic regression

Factors	*B*	Wald	OR (95% CI)	*P*
Age	1.190	2.804	3.289 (0.816–13.247)	0.094
f/t ratio	− 1.771	6.483	0.170 (0.044–0.655)	0.011
miRNA-222	− 4.331	35.166	0.013 (0.003–0.055)	< 0.001
miRNA-1248	− 1.526	5.070	0.217 (0.058–0.821)	0.024
Constant	2.622	9.437		0.002

### Cell experiment

#### Expression of miRNA-222 in PC3 and wpmy-1 cells

**Fig. 5:**
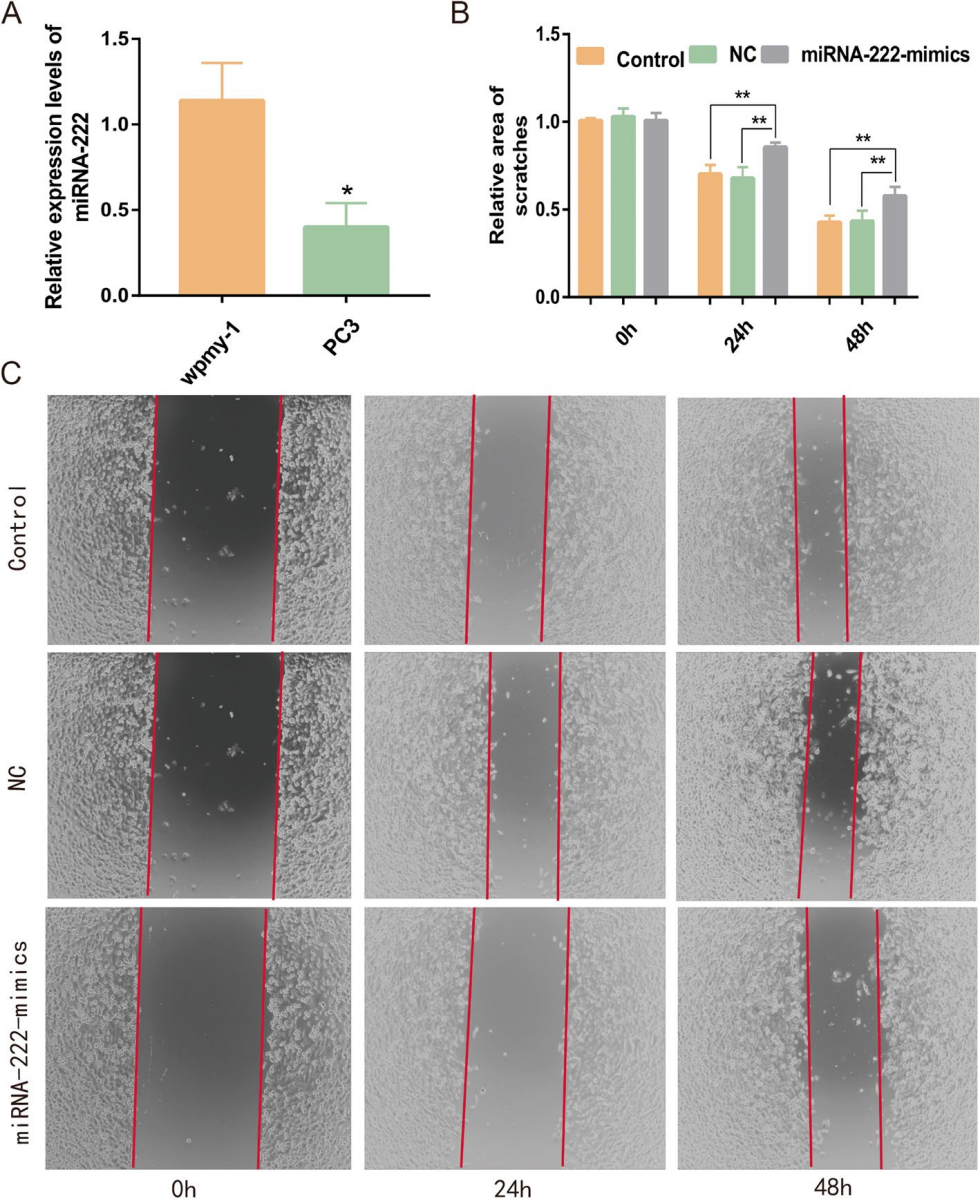
**A** Differential expression of miRNA-222 in wpmy-1 normal cells and PC-3 prostate cancer cells, * *P* < 0.05. **B** Relative scratch area of cells in different groups, ** *P* < 0.01. **C** Scratch area map of different groups of cells

In the previous study, it was found that only miRNA-222 expression in blood samples and tissue samples matched with microarray results, and the results of miRNA-222 expression in blood samples showed the largest fold relationship between the prostate cancer group and prostate hyperplasia group, therefore, the role of miRNA-222 in prostate cancer was further investigated at a later stage. We examined the expression of miR-222 in wpmy-1 and PC-3 cells using the qRT-PCR technique. The results showed that the expression of miRNA-222 in PC-3 cancer cells (0.40 ± 0.14) was significantly lower than that in wpmy-1 normal cells (1.14 ± 0.22) (*P* < 0.05) (Fig. 5A).

#### Results of transfection of PC-3 cells with miRNA-222-mimics

After transfection of PC-3 cells, qRT-PCR assay was used to verify the transfection effect after transfection, and the results showed that the expression of miRNA-222 in the cells of NC group and control group was statistically lower than that in the cells of miRNA-222-mimics group (*P* < 0.01). There was no significant difference between the expression of miRNA-222 in the cells of the NC group and control group (*P* > 0.05) (Table 12). It suggested that the miRNA-222 overexpression cell model was successfully constructed, and subsequent experiments for cell biological function detection could be performed.

**Table 12:** Transfection effect of miRNA-222

Different groups of cells	miRNA-222	*F*	*P*
Control	1.07 ± 0.06^a^		
NC	1.16 ± 0.20^a^	157.161	0.001
miRNA-222-mimics	699.52 ± 78.79^b, c^		

^a^Compared to the miRNA-222-mimics group (*P*<0.01)

^b^Compared to the blank group (*P*<0.01)

^c^Compared to the NC group (*P*<0.01)

#### Effect of overexpression of miRNA-222 on the migration ability of PC-3 cells

The scratch area of cells in the control group at 0 h was calculated using Image J software as a reference value and the relative areas of the remaining groups at different time periods were calculated. The results showed that there was no statistical difference between the relative area of scratches in the control (0.70 ± 0.05) and NC (0.68 ± 0.06) groups at 24 h (*P* > 0.05). The relative area of scratches of cells in the miRNA-222-mimics group at 24 h (0.86 ± 0.03) was greater than the area of the control and NC groups (*P* < 0.05). There was no statistical difference between the relative area of scratches in the control (0.42 ± 0.04) and NC (0.43 ± 0.06) groups at 48 h (*P* > 0.05). The relative area of scratches in the cells of the miRNA-222-mimics group at 48 h (0.58 ± 0.05) was larger than that of the control and NC groups (*P* < 0.05) (Fig. 5B, C). It was suggested that overexpressed miRNA-222 could effectively inhibit the migration ability of PC-3 cells.

#### Effect of overexpression of miRNA-222 on the proliferative capacity of PC-3 cells

**Fig. 6:**
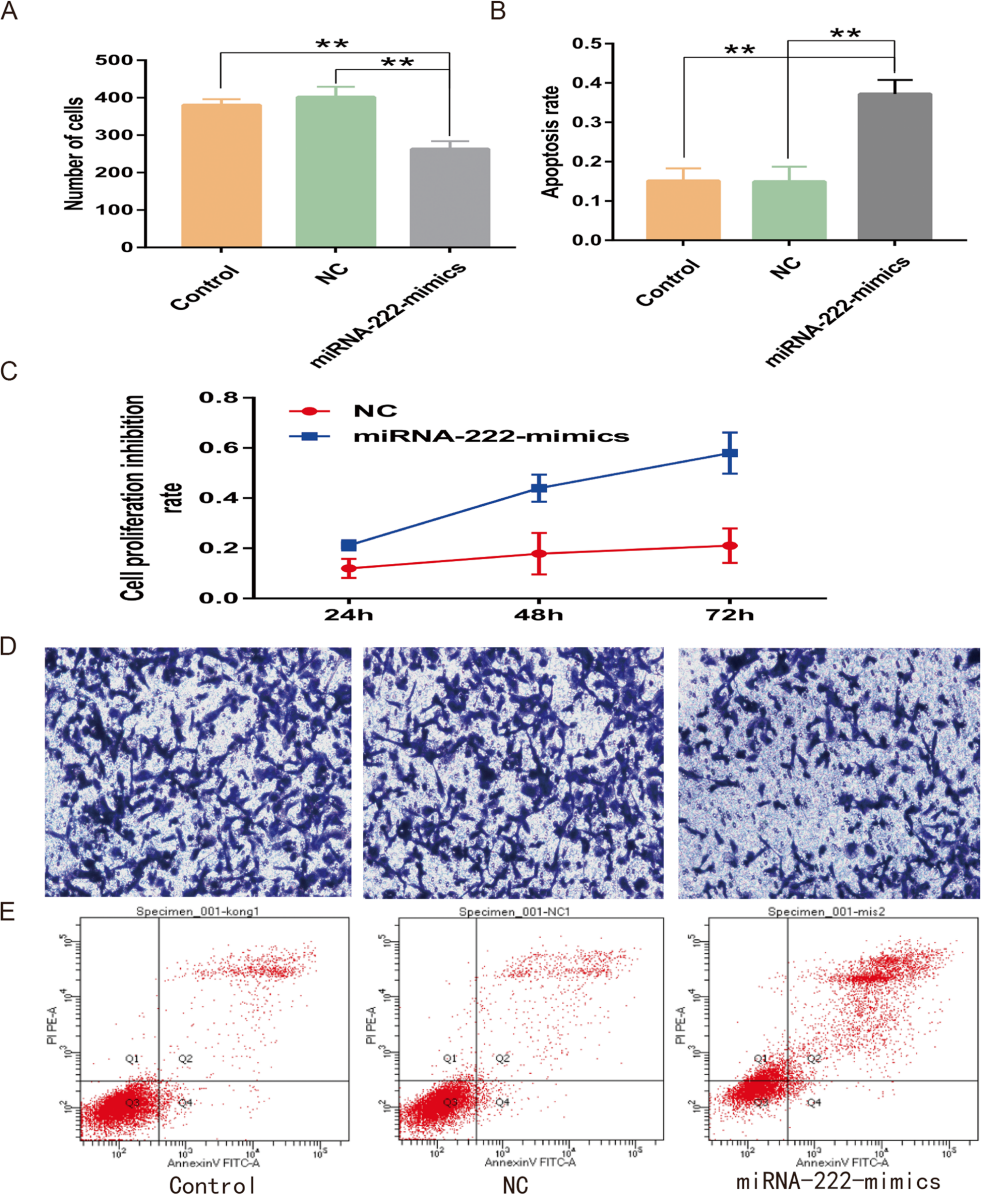
**A** Number of infestations in different groups in Transwell infestation assay, ** *P* < 0.01. **B** Apoptosis levels in different groups of cells, ** *P* < 0.01. **C** Proliferation inhibition rate of different groups of cells. **D** Results of Transwell invasion assay on different groups of cells. **E** Apoptosis levels in different groups of cells

The results of the CCK-8 assay showed that the proliferation inhibition rate of miRNA-222-mimics group cells, at 24, 48, and 72 h, was higher than that of NC group cells (*P* < 0.05), respectively (Table 13, Fig. 6C). It was suggested that overexpressed miRNA-222 could effectively inhibit the proliferation rate of PC-3 cells.

**Table 13:** Proliferation inhibition rate of cells in each group at different time periods (%)

Time (h)	NC	miRNA-222-mimics	*t*	*P*
24	11.97 ± 3.79	21.19 ± 2.03	− 4.288	0.005
48	17.85 ± 8.26	43.96 ± 5.39	− 5.296	0.002
72	21.03 ± 6.87	57.94 ± 8.22	− 10.892	0.000

#### Effect of overexpression of miRNA-222 on the invasive ability of PC-3 cells

Using Image J software to count the successfully invaded cells in the three groups of cells after 36 h, the results showed that there was no statistical difference between the number of successfully invaded cells in the control (380.67 ± 15.50) and NC (402.00 ± 27.51) groups of cells (*P* > 0.05), and the number of successfully invaded cells in the miRNA-222-mimics group of cells (263.00 ± 21.00) was smaller than the number of successfully attacked cells in control and NC group cells (*P* < 0.05) (Fig. 6A, D). It was suggested that overexpressed miRNA-222 could effectively inhibit the invasive ability of PC-3 cells.

#### Effect of overexpression of miRNA-222 on apoptosis of PC-3 cells

The apoptosis rate of cells can be obtained by calculating Q2 + Q4 in the flow cytometry assay results, and the results showed that there was no statistical difference between the apoptosis rate of cells in the control (15.13 ± 3.2) and NC (14.93 ± 3.8) groups (*P* > 0.05), and the apoptosis rate of cells in the miRNA-222-mimics group was (37.25 ± 3.54) greater than that of cells in control and NC groups (*P* < 0.05) (Fig. 6B, E). It was suggested that overexpression of miRNA-222 could effectively increase the apoptosis level of PC-3 cancer cells.

## Discussion

Most prostate cancer patients are already in an advanced cancer stage or even metastatic state at the time of initial diagnosis [10], and the treatment options for advanced prostate cancer are limited all patients turn drug resistant after a period of treatment, resulting in a poor prognosis for prostate cancer patients [11]. Early detection of prostate cancer is of great significance for prostate cancer treatment and prognosis. therefore, more accurate detection indexes for prostate cancer screening should be studied to achieve strategies such as early detection and early treatment of prostate cancer as a way to improve the early detection rate and survival of prostate cancer patients and to improve the prognosis of prostate cancer patients [12].

The role played by miRNAs as a non-coding RNA in tumors has become a hot research topic in recent years [13]. Starting with the first discovered miRNAs-lin-4 in 1993, many researchers subsequently started to explore the generation, structure, and function of miRNAs. Studies on a variety of tumors such as chronic lymphocytic leukemia, breast cancer, colorectal cancer, and lung cancer revealed that miRNA expression is abnormal in most tumor cells [14]. It is believed that miRNAs can play an important role in the diagnosis of many tumors [15]. It has been found that miRNAs are closely related to the occurrence and progression of prostate cancer, and it is believed that miRNAs are important in both the early diagnosis and malignant progression of prostate cancer [16]. Therefore, in this study, we screened miRNAs with differential expression in prostate cancer by miR microarray, and verify the potential of the miRNAs in prostate cancer diagnosis and explore the role of the validated miRNAs in the malignant progression of prostate cancer to open a new path for miRNA-based treatment of prostate cancer.

### Prostate cancer risk factors and biomarkers

The gold standard for the diagnosis of prostate cancer is puncture biopsy of prostate tissue, but puncture biopsy is a costly and invasive test, which has an impact on patients’ lives [17]. In fact, in case of transrectal biopsy, the risk of complications requiring hospital admission ranges from 0.1 to 2.5% [18] being in most of the cases secondary to urinary tract infection (UTI), fever or sepsis. Loeb et al. [19] reported a cumulative increase in the risk of having a complication where each additional biopsy was associated with a 1.7-fold increase in overall hospitalizations and a 1.7-fold increase in serious infectious complications. Clinical complications and hospital admissions following transrectal prostate biopsy have increased during the last years primarily due to an increasing rate of infections [20]. Carignan et al. [21] in 5.798 submitted to transrectal prostate biopsy demonstrated an increased incidence of infections from 0.52% in 2002–2009 to 2.15% in 2010–2011. Pietro Pepe et al. [22] retrospectively evaluated the clinical complications of 8500 patients who underwent prostate biopsy in more than 20 years of clinical practice and found that clinical complications followed by prostate biopsy biologically involved 35.9% of the patients. Urinary tract infection with over was the most frequent cause of hospital recovery (33.4% of the cases). Loeb et al. [23] in a random sample of Medicare participants in Surveillance, Epidemiology, and End Results (SEER) regions from 1991 to 2007 found that prostate biopsy was associated with a 2.65-fold increased risk of hospitalization secondary to infections within 30 days compared to the control population. Therefore, there is a need to accurately include patients for puncture biopsy. Currently, the indications for prostate puncture biopsy are mainly PSA, rectal examinations, and imaging examinations, among which PSA, as a clinically common prostate cancer-specific marker, is widely used in the fields of early tumor diagnosis, prediction of recurrence, and clinical monitoring [24]. However, the indications for prostate puncture biopsy in clinical practice are still controversial. Since 1997, Catalona et al. [25] advocated prostate biopsy for men with a PSA value in the 2.5- to 4.0-ng/ml range, believing that the use of this parameter increases the detection rate of curable forms of PCa. More recently [26], they reported on 6691 men who underwent PSA-based screening for PCa, using a mathematical model to adjust for verification bias, they estimated that 82% of the cancers in younger men and 65% of those in older men would be missed with a PSA cutoff value of 4.1 ng/ml. The considerable number of clinically significant cases with PSA ≥ 4 ng/ml has led to suggest that the PSA cut-off value should be lowered to 2.5 ng/ml in order to increase its sensitivity [27], although this increases the risk of overdiagnosis [28]. F. Aragona et al. [29] found that when PSA ≥ 10 is used as the diagnostic standard for prostate cancer, it can only detect 48.2% of prostate cancer patients. If PSA ≥ 4 is used as the diagnostic standard for prostate cancer, it can detect 91.2% of prostate cancer patients, but its specificity for diagnosing prostate cancer is particularly low. Some studies [30] have shown that elevated peripheral circulating PSA concentrations are not only seen in prostate cancer, but they may also be caused by the effects of indwelling catheterization, urethral manipulation, prostatitis, and urinary tract infection. Therefore, the specificity and sensitivity of PSA in the peripheral circulation for the diagnosis of prostate cancer is poor, and it is prone to misdiagnosis and underdiagnosis, which affects the early diagnosis and treatment of prostate cancer. Digital rectal examination is also an important method in the examination of prostate cancer, especially for asymptomatic prostate cancer patients, which is of great significance in the diagnosis and staging of prostate cancer. However, a digital rectal examination is difficult to reach tumors in the central and transitional regions, especially smaller tumor lesions, and has strong subjectivity. Due to the fact that rectal digital examination can only detect changes in prostate volume and larger palpable tumor masses, 80–90% of prostate cancer detected are advanced prostate cancer in T3 and T4 stages, and the accuracy of reported results is inconsistent. Aragona et al. [29] found that out of 36 patients with abnormal rectal digital examination, only 8 (22.8%) were diagnosed with prostate cancer, out of 779 patients with abnormal rectal digital examination and PSA, 589 (75.6%) were diagnosed with prostate cancer. Some studies suggest that for early prostate cancer with a PSA of 2.5–10 ng/ml, rectal digital examination is not correlated with prostate biopsy and pathological staging. Multi-parameter magnetic resonance imaging technology and other imaging examinations have the ability to perform multi-parameter, multi-sequence, and non-invasive imaging and have been widely applied in the detection and evaluation of prostate cancer. A study found that the detection rate for prostate cancer [31] increased with the use of mpMRI reducing the risk of overdiagnosis in comparison with systematic prostate biopsy (17 vs. 28%). The use of mpMRI in clinical practice allowed to reduce the number of needle biopsy cores performed during prostate biopsy, the reduction of needle cores could reduce prostate biopsy complications. A study [22] showed that complications following transrectal prostate biopsy were directly correlated with the number of needle cores resulting equal to 17.4% (235 cases), 38.7% (1.751 cases), and 55.3% (1.455 cases) in patients who underwent 12 vs. 18 vs. > 24 cores (*p* = 0.001), respectively; however, Hajdinjak [32] and Borofsky et al. [33] found that mpMRI can also miss clinically significant prostate cancer and may underestimate the volume of prostate cancer tissue. Underestimating the size of the lesion is also a serious issue, especially when doctors rely solely on targeted puncture results for nerve preservation surgery, which may result in residual tumors or positive margins. In addition, mpMRI single fixation of the lesion cannot guarantee that the rest of the prostate is tumor free. Therefore, in addition to PSA, rectal digital examination, and imaging examination, it is necessary to discover new indications for prostate puncture biopsy, to screen patients suitable for prostate puncture biopsy, and to reduce the harm caused by overtreatment.

The results of this study showed that f/t ratio ≤ 0.18, miRNA-222 ≤ 0.69, and miRNA-1248 ≤ 0.94 are independent risk factors for prostate cancer, and it is considered that f/t ratio ≤ 0.18, miRNA-222 ≤ 0.69, and miRNA-1248 ≤ 0.94 can be used as one of the reference indicators for patients before puncture biopsy, when patients are stratified into low-risk and high-risk by these factors. High-risk patients can be considered for puncture biopsy, and low-risk patients can avoid unnecessary invasive tests such as puncture biopsy to reduce the impact of puncture biopsy on patients’ lives to some extent.

The f/t ratio refers to the ratio of free prostate-specific antigen to total prostate-specific antigen. Previous studies have shown [34] that the risk of prostate cancer is negatively correlated with the f/t ratio and increases with a decrease in the f/t ratio. A study [35] showed that patients with f/tPSA < 0.2 were 3.84 (1.28–11.56) times more likely to develop prostate cancer than those with f/tPSA > 0.2. It has been shown [36] that f/tPSA < 0.16 is an independent influence on prostate cancer, when 0.16 was used as a diagnostic criterion for prostate cancer, the sensitivity and specificity of f/t ratio were 71.64% and 67.57%. The results of this study are consistent with them, and patients with f/t ratio < 0.18 are more likely to develop prostate cancer than those with f/t ratio > 0.18.

miRNA-222 is a member of the miRNA family, which plays different roles in different cellular microenvironments and tumors [37–40]. Most studies suggest that miRNA-222 is a “cancer-promoting factor” that plays a pro-cancer role in different tumors. miRNA-222 expression levels are significantly elevated in colon, kidney, gastric, breast, pancreatic, bladder, liver, and multiple myeloma tumors [41–46]. Although miRNA-222 plays a pro-cancer role in most tumors, it has also been reported that miRNA-222 expression levels are significantly reduced in certain tumor tissues and play a cancer-suppressive role. Liu et al. [47] found that overexpression of miRNA-222 inhibited metastasis and invasion of tongue squamous carcinoma cells and played a cancer-suppressive role. O’Hara et al. [48] found that miRNA-222 expression levels were downregulated in Kaposi’s sarcoma and exudative lymphoma. The results of the present study are consistent with them, and patients with low miRNA-222 expression levels were more likely to develop prostate cancer than those with high miRNA-222 expression levels. Zohreh Heydari et al. [49] found in their latest study that miRNA-222 was significantly upregulated in the circulating plasma of prostate cancer patients, but not significantly upregulated in prostate hyperplasia patients. The results of our study are the opposite, possibly because prostate biopsy may affect the expression of miRNA in the peripheral blood circulation. A study comparing the expression profiles of miRNAs in the circulation before and after tumor resection in lung squamous cell carcinoma found that the expression levels of miRNA-205 in plasma significantly decreased 7–10 days after tumor resection [50]. Analysis shows that among 46 miRNAs with different expressions in peripheral blood monocyte of non-small cell lung cancer patients, 42 miRNAs are down regulated after tumor resection of lung cancer [51]. It is controversial that some scholars have reported only slight differences in the preoperative and postoperative levels of miRNA-34a in non-small cell lung cancer, with let-7c expression levels increasing after surgery, while miRNA-202 and miRNA-769p showed no significant changes before and after surgery. Follow-up observation of miRNA expression in the plasma of lung cancer patients was conducted from preoperative to postoperative 18 months, it was found that the miRNA expression profile showed specific fluctuations, and the level of miRNA expression was correlated with postoperative time [52]. Therefore, these studies suggest that miRNAs in the circulation may originate from the release of tumor cells [53]. However, the source of miRNA in peripheral blood is not yet fully understood. In our study, the subjects were all divided into groups after the results of the prostate biopsy were obtained, and then blood was drawn to test the expression level of miRNA-222. Other studies may include patients who underwent prostate biopsy before taking blood to test the expression of miRNA-222, some patients who met the inclusion criteria first take blood samples and undergo prostate biopsy before grouping, without considering the changes in miRNA-222 expression before and after biopsy. Other reasons for the differences in the results of these studies may be that some patients with different disease progression may be included as the research subjects, and the miRNA expression levels of patients with different disease progression may also be different. Michele Salemi et al. [54] found an increased expression of miRNA-132 and miRNA-212 in the index case of prostatic adenocarcinoma compared to normal prostate tissue and a lower expression of miR-132 and miR-212 in metastatic lymph nodes compared to primitive PCa and normal prostate tissue. Furthermore, some limitations in our work might influence the results, which may reduce the credibility of our findings, including the low sample size, and one center sampling. Further studies are required to investigate the diagnostic value of plasma miRNA-222-3p in prostatic hyperplasia and prostatic cancer patients at a larger scale, combined with PSA as a known marker for prostatic cancer. There are also reports that miRNA-222-3p is downregulated in metastatic prostate cancer tissue compared to local prostate cancer tissue [55]. However, another study showed that miRNA-222-3p was upregulated in metastatic prostate cancer tissue [56]. Therefore, the research results of miRNA-222 in prostate cancer are still contradictory. In addition, the release control of miRNA-222-3p in the body fluids (serum, plasma, and urine) of prostate cancer patients is still unclear, and there are still some unresolved issues.

miRNA-1248 is also a member of the miRNA family, and it plays different roles in different tumors [57]. Yuhao et al. [58] found that miRNA-1248 was significantly different in intrahepatic cholangiocarcinoma tissues and paraneoplastic tissues by miRNA microarray, and qRT-PCR was used to detect the expression of miRNA-1248 in 139 cancer and normal patient tissues, verifying that miRNA-1248 expression was upregulated in intrahepatic cholangiocarcinoma tissues. Tanic M et al. [59] found that miR-1248 can be used as a genetic test standard in hereditary breast cancer. A study [60] showed that miR-1248 could inhibit the proliferation of gastric cancer cells and induce apoptosis of gastric cancer cells for cancer suppression purposes. Xiaoyuan et al. [61] found that the expression of miRNA-1248 in the plasma of liver cancer and non-hepatocellular carcinoma patients differed by high-throughput sequencing. qRT-PCR was used to detect the expression of miRNA-1248 in the plasma of 139 cancer and normal patients, and it was verified that miRNA-1248 expression was down-regulated in the plasma of liver cancer patients. The results of this study were consistent with it, and patients with low miRNA-1248 expression levels were more likely to develop prostate cancer than those with high miRNA-1248 expression levels.

### Regulation of prostate cancer by miRNA-222

It has been found that certain miRNAs can promote or inhibit the metastatic and invasive ability of tumor cells. Also, some miRNAs affect the apoptosis of tumor cells by promoting or inhibiting the proliferation ability of cells. It plays a crucial role in the malignant progression of prostate cancer [62]. It has been reported that antagonists and agonists of miRNAs have been synthesized to affect the biological function of prostate cancer cells by blocking or restoring the function of specific miRNAs [63]. Alternatively, miRNA or anti-miRNA molecules can be delivered into the body through a vector vehicle, and these small molecules can induce changes in the biological functions of the targeted cells by systemic or local administration. If these antitumor molecules specifically alter the biological specificity of tumor cells, then these molecules may be harmless to normal cells. The application of these antitumor molecules in vivo opens new paths for miRNA-based therapy for prostate cancer.

In this study, miRNA microarray technology was used to identify 13 miRNAs that differ in prostate cancer tissues from non-prostate cancer tissues. By reviewing the literature, we selected four known miRNAs that have rarely been studied in prostate cancer (hsa-miRNA-1248, hsa-miRNA-144-3p, hsa-miRNA-3651, hsa-miRNA-222), the present study found that the expression of hsa-miRNA-222 among the above four miRNAs was consistent in microarray results, tissue samples, and blood samples. In addition, although miRNA-222 has been shown to be one of the cancer suppressor or cancer-promoting miRNAs in malignant tumors such as colon cancer, kidney cancer, gastric cancer, breast cancer, pancreatic cancer, bladder cancer, liver cancer, multiple myeloma, tongue squamous carcinoma, Kaposi's sarcoma and exudative lymphoma, its role in prostate cancer is unclear. Bin Gui et al. [64] found in their latest study that miRNA-222, as a carcinogenic gene for prostate cancer, promotes the proliferation of prostate cancer cells and the development of castration-resistant prostate cancer (CRPC) in the early stages, indicating that miRNA-222 has a cancer-promoting effect. Other studies have shown that miRNA-222 expression is downregulated in metastatic prostate cancer and CRPC specimens, indicating that miRNA-222 has tumor-inhibitory effects [65–67]. The expression and biological function of miRNA-222 in prostate cancer are still controversial. Therefore, in this study, miRNA-222 was used as a research target to explore the regulation of miRNA-222 in prostate cancer and to provide a new basis for miRNA-222-based treatment of prostate cancer.

Previous studies have indicated that miRNA-222 has a complex function in different tumors and has important applications in the early diagnosis, treatment, and prognosis prediction of malignant tumors. Overexpressed miRNA-222 is associated with invasive ability, apoptosis, and cell proliferation in a variety of tumor cells [43, 44]. It was found that upregulation of miRNA-222 expression can cause increased invasion, growth, and metastatic ability of tumor cells and even lead to conditions such as drug resistance [68, 69]. Zhang et al. [70] found that overexpressed miRNA-222 in gastric cancer increased the invasion and proliferation ability of gastric cancer cells by regulating the expression of PTEN. It was also found that miRNA-222 overexpressed in gastric cancer cells increased the proliferative ability of gastric cancer cells by regulating the expression of RECK [71]. The above studies suggest that miRNA-222 expression in gastric cancer may become a biomarker and target for targeted therapy in gastric cancer. Liu et al. [47] found that miRNA-222 overexpressed in tongue squamous cell carcinoma exerted cancer-suppressive effects by reducing the expression of peroxisome 2 and matrix metalloproteinase 1 and inhibiting the metastatic and invasive ability of cells. The results of the present study are consistent with this finding. The invasive, proliferative, and migratory abilities of miRNA-222 overexpressed PC-3 cells were significantly inhibited, and the apoptosis rate was significantly increased. It can be concluded that miRNA-222 plays an important role in inhibiting the malignant progression of prostate cancer, which provides a new direction and basis for the study of new therapeutic targets and new biomarkers for prostate cancer.

### Innovations in research

In this study, we compared the expression of four miRNAs (hsa-miRNA-1248, hsa-miRNA-144-3p, hsa-miRNA-3651, and hsa-miRNA-222) that have not been studied in prostate cancer in Xinjiang prostate cancer patients and prostate hyperplasia patients. Although there are more studies related to these miRNAs in other malignancies, most of them mainly compare the expression of miRNAs in tumor and non-tumor tissues. In this study, in order to control for the bias that prostate hyperplasia also leads to miRNA expression, we mainly compare the expression of miRNAs in tissues and blood of patients with prostate cancer and prostate hyperplasia and find that these miRNAs play a role in prostate cancer diagnosis and inhibition of malignant progression.

### Supplementary Information

**Additional file 1: eTable 1.**  Comparison of Small RNA sequences in prostate cancer tissue and prostate cancer hyperplasia tissue with the Rfam database.

## Data Availability

We declare that the data and materials in this study will be provided free of charge to scientists for noncommercial purposes. All authors ensure that our data does not contain any of the following: (a) Infringes or makes unauthorized use of the Intellectual Property Rights. (b) Any other right of any person. (c) Is defamatory, derogatory, discriminatory, or violates any rights of privacy. (d) Breaches any applicable law or regulation. (e) Contains a virus, malware, or other potentially harmful components, information, or instructions.
