# Supplementary material for: Preliminary study on miRNA in prostate cancer
Source: World J Surg Oncol. 2023 Aug 29;21:270. doi: 10.1186/s12957-023-03151-1 (PMC10464187; doi:10.1186/s12957-023-03151-1)
Supplement: Supplementary file 1 — Additional file 1: eTable 1. Comparison of Small RNA sequences in prostate cancer tissue and prostate cancer hyperplasia tissue with the Rfam database. [file 12957_2023_3151_MOESM1_ESM.doc]

eTable 1 Comparison of Small RNA sequences in prostate cancer tissue and prostate cancer hyperplasia tissue with the Rfam database

| Category | Ca2 | |  | Ca4 | |  | Ca5 | |  | Ca14 | |  | N7 | |  | N11 | |  | N15 | |  | N16 | |
| --- | --- | --- | --- | --- | --- | --- | --- | --- | --- | --- | --- | --- | --- | --- | --- | --- | --- | --- | --- | --- | --- | --- | --- |
|  | Number of sequences | Percentage |  | Number of sequences | Percentage |  | Number of sequences | Percentage |  | Number of sequences | Percentage |  | Number of sequences | Percentage |  | Number of sequences | Percentage |  | Number of sequences | Percentage |  | Number of sequences | Percentage |
| Pure Sequence | 11 827 333 | 100.00 |  | 14 950 214 | 100.00 |  | 13 475 826 | 100.00 |  | 10 772 859 | 100.00 |  | 14 448 964 | 100.00 |  | 17 452 607 | 100.00 |  | 13 662 588 | 100.00 |  | 12 693 394 | 100.00 |
| Contrasting successful sequences | 9 275 678 | 78.55 |  | 11 101 410 | 74.38 |  | 9 510 899 | 70.70 |  | 7 842 534 | 72.92 |  | 12 112 762 | 83.97 |  | 13 012 039 | 74.68 |  | 9 247 447 | 67.80 |  | 9 208 898 | 72.67 |
| rRNA | 698 054 | 5.91 |  | 1 024 560 | 6.86 |  | 1 293 731 | 9.62 |  | 951 662 | 8.85 |  | 663 294 | 4.60 |  | 1 229 786 | 7.06 |  | 1 286 890 | 9.43 |  | 1 032 836 | 8.15 |
| tRNA | 127 117 | 1.08 |  | 182 016 | 1.22 |  | 258 665 | 1.92 |  | 141 871 | 1.32 |  | 102 868 | 0.71 |  | 361 185 | 2.07 |  | 78 902 | 0.58 |  | 138 163 | 1.09 |
| snRNA | 162 819 | 1.38 |  | 407 028 | 2.73 |  | 226 636 | 1.68 |  | 185 083 | 1.72 |  | 111 285 | 0.77 |  | 254 550 | 1.46 |  | 12 0617 | 0.88 |  | 178 821 | 1.41 |
| Cis-reg | 15 356 | 0.13 |  | 35 047 | 0.23 |  | 51 186 | 0.38 |  | 67 833 | 0.63 |  | 29 025 | 0.20 |  | 49 861 | 0.29 |  | 58 623 | 0.43 |  | 33 249 | 0.26 |
| miRNA | 817 7526 | 69.25 |  | 9 248 865 | 61.97 |  | 7 456 240 | 55.42 |  | 6 411 109 | 59.61 |  | 11 124 706 | 77.12 |  | 10 887 158 | 62.49 |  | 7 606 199 | 55.76 |  | 7 737 038 | 61.06 |
| others | 94 806 | 0.80 |  | 203 894 | 1.37 |  | 224 441 | 1.67 |  | 84 976 | 0.79 |  | 81 584 | 0.57 |  | 229 499 | 1.32 |  | 96 216 | 0.71 |  | 88 791 | 0.70 |
